# A Single Posterolateral Scapular Approach to Drain Post-Traumatic Intramuscular Scapular and Axillary Abscess in an Adolescent: A Case Report

**DOI:** 10.5704/MOJ.1907.012

**Published:** 2019-07

**Authors:** YC Khaw, WI Faisham

**Affiliations:** Department of Orthopaedics, Universiti Sains Malaysia, Kubang Kerian, Malaysia

**Keywords:** scapula abscess, axillary abscess, adolescent

## Abstract

Scapular abscess is a rare clinical diagnosis. This is a report of an atypical case of extensive intramuscular scapular abscess involving the anterior and posterior aspects of the scapula with extension into the axillary region following minor trauma in a young healthy adolescent, describing a single posterolateral approach to the scapula to evacuate the abscess. Following surgical drainage and antibiotic treatment, patient recovered without any complication.

## Introduction

Scapular abscess is an uncommon clinical diagnosis. A literature search yielded only 11 cases being reported so far^1-5^; and mostly of subscapular abscess. Early diagnosis, urgent surgical intervention and antibiotic treatment are warranted for scapular abscess. Any delay in diagnosis or treatment may progress to septicaemia and may even lead to death^1^. A high index of suspicion is required to diagnose scapular abscess as it is rarely encountered in clinical practice. We report the case of a young healthy adolescent with extensive intramuscular scapular abscess extending into the axillary region following a minor trauma.

## Case Report

**Fig. 1:**
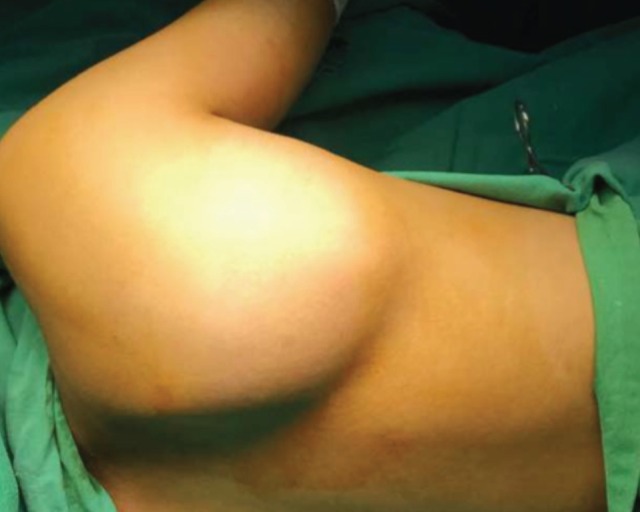
Swelling at right scapular region.

A 13-year old girl presented with right shoulder pain associated with fever for nine days. The problem started when her right upper limb was pulled by her brother to drag her out of bed. Pain and swelling at her right scapular region (Fig. 1) progressively increased in size since the incident. She was treated as soft tissue injury with NSAIDS initially.

Three days later, her symptoms worsened and she started to have low-grade fever. She underwent traditional massage twice for her symptoms. She was prescribed oral cloxacillin by a general practitioner; however, her symptoms became worse, with loss of appetite and weight of 2 kg within a week.

Upon admission, her pulse rate was 125 beats/minute, blood pressure was 110/69 mmHg with temperature of 37.4°c. Examination revealed, a swelling at the right scapular region, measuring 5cm x 7cm, tender, warm and fluctuant and she was unable to move her right shoulder due to pain. The white blood cell count was 27 x 10^3^/μL, erythrocyte sedimentation rate was 87 mm/hr, and C-reactive protein was 106 mg/L. Blood culture showed no growth.

**Fig. 2:**
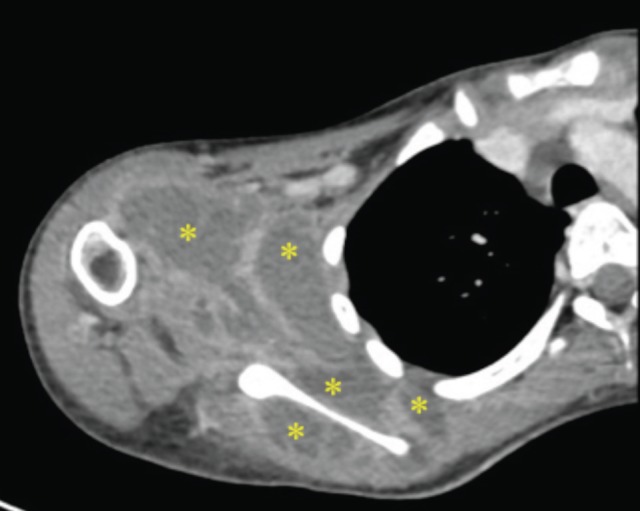
Computer Tomographic scan revealing multiloculate intramuscular abscess (yellow ^_*_^ ) at right anterior and posterior scapula extending into axillary region in the axial view.

Ultrasound showed multi-loculated intramuscular collections around the right scapula. Computer Tomographic scan (Fig. 2) revealed multiple multi-loculated intramuscular abscesses at right anterior and posterior scapula extending into the axillary region, involving subscapularis, infraspinatus, teres minor and serratus anterior muscles. The shoulder joint was not involved.

**Fig. 3:**
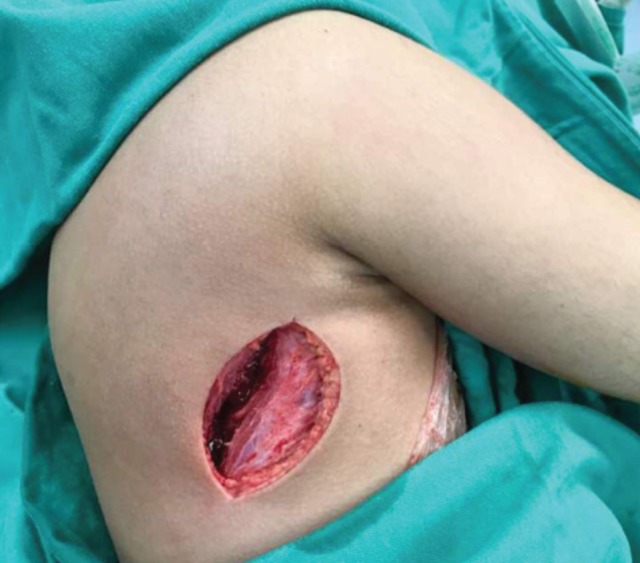
A single posterolateral surgical approach used to drain scapular and axillary abscess.

A single posterolateral surgical approach (Fig. 3) was used to drain the entire multi-loculated abscess. The lateral wall of the scapula was approach by detaching the teres minor and major muscles and subsequently draining the abscess over the subscapularis space and the thoracic cage. The abscesses anterior to the infraspinatus muscle was drained by elevation of muscle from the lateral scapular border. The pus culture revealed *Staphylococcus aureus*, sensitive to cloxacillin. She completed three weeks of oral cloxacillin and the wound closed by secondary intention. She had regained full range of shoulder motion and radiograph at three months showed no osteomyelitis changes of the scapula.

## Discussion

The scapular region is not a common location for abscess formation. Thus far, there are only 11 cases of scapular abscess reported^1-5^ in the literatures and mainly located around the subscapular region. Our case demonstrates a more extensive abscess involving the entire periscapular region and extending to the thoracic space. Such an extensive abscess involving the scapula and axillary regions has not been reported in literature. We postulate that local pressure with massage had breached the anatomical barrier leading to extension of the abscess to a nearby compartment. Systemic infection, immune-compromised diabetes and trauma had been reported to cause scapular abscess^1-5^. Minor trauma in our case might cause hematoma formation in scapular region leading to formation of scapular abscess. Handorf *et al*^1^ reported a 19-year old male who had developed subscapular abscess after the shoulder was hit by baseball bat and Babayigit *et al*^2^ reported a case of subscapular abscess in a 7-year old boy after falling off a bicycle and sustaining trauma to the left shoulder. All the cases highlighted the possibility that minor trauma with soft tissue injury and hematoma could be the cause of the scapular abscess.

Young patients presenting with painful shoulder swelling and fever can mislead with the diagnosis of septic arthritis or acute osteomyelitis of the proximal humerus, which are common in children. Tuberculosis of the joint should be considered in the differential diagnosis as tuberculosis is common in our country. It is challenging to differentiate all these differential diagnoses with scapular abscess, therefore a high index of suspicious with good history taking and physical examination are needed. In our case, ultrasound revealed multi-loculated intramuscular collection around the right scapula not involving the shoulder joint, thus the need for joint aspiration was excluded. Although scapular abscess is a rare diagnosis, delay in diagnosis and treatment may lead to septicaemia and death. Handort *et al*^1^ reported the case of a 9-year old child’s shoulder was hit by a baseball bat, led to septicaemia and death, the subscapular abscess being identified at autopsy.

CT scan helps to support the diagnosis of abscess and evaluate the anatomical area and extent of the abscess. Planning for surgical approach in this complex anatomical area is important to minimise the surgical damage and evacuate the loculated abscess completely. To our knowledge, only four case reports of subscapular abscess describe the surgical approach to drain subscapular abscess: vertical incision along medial border of scapula^2^, anterior deltopectoral approach^3^, modified Judet approach^4^, and posterolateral approach to scapula with medial counter incision^5^. The single posterolateral incision was used in our case because the approach was easy and we were able to drain out the extensive periscapular abscess. This single approach did not need additional medial incision.

In conclusion, scapular abscess is rare and challenging to diagnose. The posterolateral approach to scapula is a simple approach and effective way to drain extensive scapular abscess. Early diagnosis, prompt antibiotic administration and surgical intervention to drain abscess is important in the treatment of scapular abscess, for the patient to recover without complications.
